# Does a specialist typeface affect how fluently children with and without dyslexia process letters, words, and passages?

**DOI:** 10.1002/dys.1727

**Published:** 2022-08-27

**Authors:** Holly Joseph, Daisy Powell

**Affiliations:** ^1^ Institute of Education University of Reading Reading United Kingdom

**Keywords:** dyslexia, eye movements, rapid naming, reading, specialist typeface

## Abstract

Children with dyslexia are at risk of poor academic attainment and lower life chances if they do not receive the support they need. Alongside phonics‐based interventions which already have a strong evidence base, specialist dyslexia typefaces have been offered as an additional or alternative form of support. The current study examined whether one such typeface, Dyslexie, had a benefit over a standard typeface in identifying letters, reading words, and reading passages. 71 children, aged 8–12 years, 37 of whom had a diagnosis of dyslexia, completed a rapid letter naming task, a word reading efficiency task, and a passage reading task in two typefaces, Dyslexie and Calibri. Spacing between letters and words was kept constant. Results showed no differences in word or passage reading between the two typesfaces, but letter naming did appear to be more fluent when letters were presented in Dyslexie rather than Calibri text for all children. The results suggest that a typeface in which letters are designed to be distinctive from one another may be beneficial for letter identification and that an intervention in which children are taught letters in a specialist typeface is worthy of consideration.

**Practitioner Points**

- Commercially available specialist typefaces claim to make reading easier and more fluent for dyslexic readers
- We examined whether presenting letters, words, and passages to children in a specialist compared to a standard typeface improved their fluency
- Results showed no difference for dyslexic or typical readers in word and passage reading
- There was a small advantage for the specialist typeface in letter naming, suggesting the typeface helped letter discrimination
- It may help some children (e.g. those at risk of dyslexia) in early years to learn letters in a specialist typeface

## 1 INTRODUCTION

Mastering the mechanics of reading is a necessity for reading comprehension and educational success. Children with a diagnosis of dyslexia experience specific difficulties with reading and spelling words accurately and fluently, posing a significant challenge to their academic attainment. Although intervention studies have shown that phonics‐based interventions are effective in improving reading outcomes for this group (Hulme, Bowyer‐Crane, Carroll, Duff, & Snowling, 2012), a number of alternative solutions, focussing on visual aspects of reading, have been offered. These include coloured overlays or glasses and specialist “dyslexia‐friendly” typefaces. The current study examined whether one such specialist typeface, Dyslexie (Boer, 2016), affected letter naming, word reading fluency, and reading times on connected text as measured using eye tracking. If something as simple as changing typeface design does improve reading efficiency at the letter, word, or sentence level, there is a clear benefit to educators, parents, and dyslexic readers.

The Simple View of Reading (Hoover & Gough, 1990) stipulates that reading comprehension is the product of two components: decoding or word recognition, and linguistic comprehension. Each is necessary but alone not sufficient for successful reading to develop. While some children, so‐called poor comprehenders (Catts, McIlraith, Bridges, & Nielsen, 2016) have specific difficulties in linguistic comprehension, children with a dyslexic profile exhibit difficulties in the decoding/word recognition component, meaning that they struggle to identify words accurately and fluently from their written form.

The definition of dyslexia has proved controversial due to ongoing debates regarding whether dyslexia is an appropriate label if a child has more general learning difficulties (Stanovich, 1991) or co‐morbidities. However, the DSM5 (American Psychiatric Association, 2013) definition is relatively uncontroversial: it describes dyslexia as a specific learning disorder which is characterised by difficulties with accurate or fluent word reading, poor decoding, and poor spelling. It is also well‐established that phonological (speech sounds) difficulties typically underlie dyslexia (Catts et al., 2016; Shankweiler, Liberman, Mark, Fowler, & Fischer, 1979; Snowling, 2000; Snowling & Hulme, 1994; Vellutino, Fletcher, Snowling, & Scanlon, 2004). Poor quality phonological representations make it harder to learn mappings between print and sound which is at the heart of decoding. However, it is also the case that dyslexia is highly likely to be multifactorial and therefore involves the interaction of multiple risk factors, not just a phonological deficit (Catts et al., 2016; Pennington, 2006).

What causes dyslexia links directly to what interventions will be most successful. The phonological deficit hypothesis (e.g., Snowling, 2000) posits that training in phonological skills (in particular phonological awareness: the ability to recognise and manipulate speech sounds) should improve reading accuracy and fluency and this has indeed proved to be the case when combined with letter‐sound training (Bradley & Bryant, 1983; Hatcher et al., 2006; Hulme et al., 2012), especially in English where the relationship between reading and phonological awareness is more enduring than in languages with more transparent orthographies (Landerl et al., 2013). In addition, the double deficit hypothesis (Wolf & Bowers, 1999) suggests that children with dyslexia may have a deficit in phonological awareness, rapid automatised naming, or both; and having both results in a more severe presentation of reading difficulties. Rapid automatised naming (RAN) tasks measure the ability to name sequentially presented letters, digits, colours, or objects as quickly as possible. While some researchers argue that RAN forms part of a broader phonological deficit (Vellutino et al., 2004), others maintain that RAN problems can exist in the absence of phonological awareness deficits in dyslexic children and thus that RAN constitutes an additional potential cause for dyslexia (Pennington et al., 2012; Wolf et al., 2002). Intervention RAN studies are less compelling than those training phonological skills although some have argued that it has a causal role in dyslexia (Vander Stappen, Dricot, & Van Reybroeck, 2020). If these early RAN training results were replicated, then we would have good reason to argue for both rapid naming and phonological awareness to form part of a successful intervention for children with dyslexia. In addition, the wide acceptance of a multifactorial model of dyslexia (Pennington, 2006) means that it is sensible to consider multiple causes which may open the door to multiple interventions.

Reading requires visual as well as phonological processing and intuitively it makes sense that reading difficulties could be caused by problems within either or both systems. Pertinent to the use of specialist typefaces for dyslexic readers is the issue of visual crowding. Visual crowding is described as difficulty identifying objects (e.g., letters) which are surrounded by similar items (e.g., other letters) as compared with when they are presented in isolation (Bouma, 1970). The evidence for increased visual crowding in dyslexia is mixed (Atkinson, 1991, 1993; Bouma & Legein, 1977; Spinelli, De Luca, Judica, & Zoccolotti, 2002) and if increased crowding does indeed occur in dyslexic readers, it is not clear whether this is a cause or a consequence of dyslexia. Notwithstanding the direction of cause, if visual crowding does occur in some dyslexic readers, then it is possible that changes in typeface, most especially increasing spacing between words, letters, and lines of text, may ameliorate reading accuracy and fluency. Indeed, previous research has shown that increasing print size (O'Brien, Mansfield, & Legge, 2005) and spacing (Duranovic, Senka, & Babic‐Gavric, 2018; Zorzi et al., 2012) results in faster and more accurate reading in children with dyslexia (although see van den Boer & Hakvoort, 2015; Łuniewska, Wójcik, & Jednoróg, 2021 for evidence to the contrary), but not consistently in typically developing children (e.g., Duranovic et al., 2018; Reynolds & Walker, 2004; Wilkins, Cleave, Grayson, & Wilson, 2009). Evidence from adults shows that increasing spacing beyond so‐called critical letter spacing (which conforms to standard spacing in most typefaces) does not increase reading speed (Chung, 2002).

In addition to spacing, typeface designs can vary on a number of other characteristics to make letters look distinctive. These include varying x‐height (the distance between the baseline and the mean line of lower‐case letters, typically the height of the letter *x*), increasing the length of descenders and ascenders (parts of letters that extend above and below the baseline or x‐height), and changing the body size or shape (for example increasing weight, adding serifs, or making text bold or italic). Given that letter identification and position coding (for example to distinguish *causal* and *casual*) is known to be a critical part of word identification (Davis, 2010), a typeface that makes letters more easily distinguishable from one another may benefit efficient word reading.

Dyslexie typeface (Boer, 2016) was created to address the difficulties outlined above: increasing spacing between letters (and words) and designing letter shapes to make them distinctive from one another was thought to reduce the likelihood of readers confusing similar letters such as *i* and *j*. This was done by changing letter heights and shapes, introducing oblique (slanting) letters, increasing openings within letters, making the base of letters thicker, and making capital letters and punctuation marks larger. In particular, having “weighted bottoms”, that is, increasing the thickness of the base a letter, was thought to reduce the likelihood of readers, especially beginning and dyslexic readers, flipping or rotating similar letters such as *b* and *p*, thus helping them to accurately identify and discriminate letters, leading to more accurate and fluent reading. Whether letter reversals and orientation errors are more common in children with dyslexia is highly disputed (Lachmann & Geyer, 2003; Terepocki, Kruk, & Willows, 2002). However, it is reasonable to argue that the typeface design changes described above may result in a reader experiencing less visual crowding and less confusion of similar letters. Whether such changes result in more accurate and fluent reading, however, requires careful empirical examination.

A number of studies have examined whether Dyslexie typeface is effective in increasing reading speed and accuracy in dyslexic readers. Marinus et al. (2016) tested 39 “low‐progress” readers (defined as those who scored below the 25th percentile in a word reading test) in Grades 2–6 on their reading fluency of four passages. There were four versions of each passage: Dyslexie font, Arial font with standard spacing, Arial font with overall matched spacing to Dyslexie, and Arial font with balanced matched spacing to Dyslexie. The difference between the latter two conditions was that in the balanced matched condition, spacing within and between words was matched to Dyslexie (with a larger increase in space between words than between letters) whereas in the overall matched condition, the increase in spacing was the same between words and between letters. Therefore, the balanced matched condition most closely approximated Dyslexie font. Results showed that the Dyslexie font passages were read faster than the unmatched and overall matched Arial passages, but there was no difference between the Dyslexie and the balanced matched Arial passages. This pattern of results shows that spacing rather than the design of the typeface per se is what drives increased reading fluency (see also Duranovic et al., 2018 for further evidence of this). As it is easy to adjust both within and between word spacing in widely available software packages such as Microsoft Word, the results of the Marinus et al. study do not support the need for specially designed typefaces for dyslexic readers.

Kuster, van Weerdenburg, Gompel, and Bosman (2018) conducted two experiments to investigate further the potential benefits of Dyslexie. In Experiment 1, 170 children, aged 7–12 years, were selected according to their performance on word reading and spelling tests: only those below the tenth percentile on either or both tests took part. Children read texts from a standardised assessment in both Arial and Dyslexie typeface (matched on size and spacing), with 2 weeks between reading of each typeface. Results showed that reading was faster at Time 2 in general but there were no significant differences in reading speed or accuracy across typefaces, showing no advantage (nor disadvantage) for Dyslexie. In Experiment 2, 147 children, aged 7–12 years, with (*n* = 102) and without (*n* = 45) dyslexia read word lists in Arial, Times New Roman and Dyslexie typefaces, matched for spacing. The two groups were matched on text reading skills, but the dyslexic group was recruited from younger grades. Children with dyslexia read more slowly overall but there was no difference between typeface types, consistent with findings from Experiment 1.

From previous research then, it appears that Dyslexie typeface per se does not carry a benefit in terms of reading fluency in dyslexic (or typically developing) children, although increasing spacing between letters and words, up to a critical threshold, does appear to be advantageous. Other studies have established that typeface design in itself does not improve reading fluency or accuracy using other “dyslexia friendly” typefaces (Galliussi, Perondi, Chia, Gerbino, & Bernardis, 2020; Wery & Diliberto, 2017), with one notable exception that did not control for spacing (Bachmann & Mengheri, 2018). However, previous studies have focused on global passage (Marinus et al., 2016), sentence (Kuster et al., 2018) and single word (Kuster et al., 2018) reading times. While this research has been extremely informative, it remains possible that overall reading times do not capture subtle differences in reading behaviour between typefaces. Another possibility is that Dyslexie typeface is beneficial at the letter level. Key theories of reading (e.g., Perry, Ziegler, & Zorzi, 2007; Rumelhart & McClelland, 1982) argue that processes underlying word recognition are highly interactive, with input from both bottom‐up (letter/feature level) and top‐down (word level) processes, as evidenced by the fact that letters are detected more quickly when embedded in words than in nonwords (e.g., STXRN) or even than when presented alone (Reicher, 1969; Wheeler, 1970). Such top‐down effects have also been shown in young, developing readers (Coch, Mitra, & George, 2012), including those with dyslexia (Grainger, Bouttevin, Truc, Bastien, & Ziegler, 2003). Thus, as studies above investigating dyslexic‐specific typefaces focussed only on word and text level reading efficiency, any effects at the level of letter processing may have been obscured by the effects of top‐down processes. If specific typefaces do make letter identification easier, this has implications for early stages of reading development as well as for reading in children with dyslexia.

The current study aimed to address these unanswered questions by examining fluency naming letters and fluency reading at the word and passage level, and using eye movements to capture subtle differences in eye movement behaviour during reading. Eye movements may uncover qualitative differences in saccades or fixations that do not equate to an overall difference in reading times. For example, children may make longer initial fixations but fewer or shorter subsequent fixations on letters in Dyslexie typeface than its standard alternative (Calibri in this experiment). Eye tracking can therefore tell us *how* a child is reading as well as how long it takes.

The experiment had a 2 × 2 × 2 design. Two typefaces, Dyslexie and Calibri were compared and this was manipulated within participants. In the passage reading task, there was a manipulation of word type: “easy‐to‐read” (more consistent grapheme‐phoneme correspondences or GPCs, for example, *beetle*) and “hard‐to‐read” (less consistent GPCs, for example, *vehicle*) and again this was manipulated within participant. In the RAN task we had a similar manipulation in which letters were classed as “regular” (less visually confusable with other le, for example, *k*) and ‘confusable’ (e.g., b). Finally, there were two participant groups: children with and without a diagnosis of dyslexia.

Our design allowed us to ask three main research questions:

1. Do children with and without dyslexia differ in their naming speed of letters in Dyslexie versus Calibri typeface, and of ‘regular’ and ‘confusable’ letters?

Because dyslexia‐friendly typefaces are designed specifically to make letters more easily distinguishable, we predicted that children with dyslexia were most likely to exhibit a Dyslexie benefit in this task. While this has not been shown at the word level, it is possible that when the task requires only letter identification and naming (i.e., top‐down [word‐level] processing does not eradicate letter‐level effects), the design of the letterform will have an effect, and that the effect is likely to be greater in children who find rapidly identifying and naming letters hard (i.e., the dyslexic group). Furthermore, we expected the Dyslexie benefit to be greater for confusable than regular letters as it would be here that differences between letter forms would matter most for the dyslexic group.

2 Do children with and without dyslexia differ in their reading efficiency of single words in Dyslexie versus Calibri typeface?

Consistent with previous research we did not expect to see any effect of typeface in the single word reading task.

3 Do children with and without dyslexia differ in their reading times on target words embedded in text as a function of typeface and difficulty?

In the passage reading task, we did not expect to observe a Dyslexie typeface benefit in global reading times for children with or without dyslexia, consistent with previous studies. However, for the reading times on target words, we predicted that a three‐way interaction between group, word type, and typeface might be apparent on hard‐to‐read words. We know that dyslexic readers may have difficulties storing orthographic representations of words, and thus might particularly struggle reading inconsistent words (Castles & Coltheart, 1993; McArthur et al., 2015; Share, 1995) and it is plausible that distinctive letterforms may disproportionately benefit dyslexic readers on these more difficult words, resulting in a smaller difference between easy‐to‐read and hard‐to‐read words in Dyslexie typeface for the dyslexic group only. In this way, the passage reading task was quite different in terms of predictions from the single word reading task, precisely because the word reading task did not contain a manipulation of orthographic difficulty.

We predicted no main effect of group in any task as we matched groups on word reading efficiency.

## 2 METHOD

### 2.1 Participants

Children were recruited from one specialist dyslexia school and one state primary school in the southeast of England. Following Marinus et al. (2016) who tested 39 children, we aimed for 40 children per group. A power analysis indicated that this was sufficient to detect a medium effect size (*d* = 0.4; approximately 50 ms). Eighty‐one children were tested but ten participants were excluded for the following reasons: did not complete all tasks (*n* = 1); did not comply with task instructions (*n* = 4); slow reading (timed out, *n* = 2); and tracker loss (*n* = 3). The remaining 71 children were split into two groups: those with a diagnosis of dyslexia (*n* = 37) and those without (*n* = 34). All children with a dyslexia diagnosis came from the specialist dyslexia school which required a diagnosis of dyslexia for admission. Children from this school were selected by the school special educational needs coordinator (SENCo) to ensure that they did not have any (known) additional diagnoses. In the state primary school, one child was excluded for having a dyslexic profile (standard score below 85 for word and nonword reading) of reading but no diagnosis. All children had normal or corrected to normal (i.e., with glasses) vision and were able to take part in the experiment. One child in the dyslexia group completed the offline tasks using a coloured (pink) overlay at their request.

Table 1 shows mean standard scores on the Test of Word Reading Efficiency (TOWRE‐2) word and nonword reading efficiency tests and on the British Picture Vocabulary Scale (BPVS). Groups were matched on word reading skill (raw score) and so differed substantially in age and vocabulary (raw score).

**TABLE 1:** Mean raw and standard scores and chronological age of children in the dyslexic and control groups. Standard deviations in parentheses

		Control	Dyslexia
Chronological age		8.0 *(0.58)*	11.09 *(1.73)*
TOWRE‐2 words (Calibri)	Raw	57 *(13)*	58 *(14)*
	Standard	107 *(12)*	83 *(15)*
TOWRE‐2 nonwords (Calibri)	Raw	31 *(12)*	27 *(11)*
	Standard	108 *(14)*	84 *(13)*
BPVS	Raw	101 *(15)*	131 *(17)*
	Standard	95 *(13)*	93 *(12)*

### 2.2 Materials

All children completed three standardised tests in addition to the main experiment. All children completed the Test of Word Reading Efficiency (TOWRE‐2; Torgesen, Wagner, & Rashotte, 1999) first. In this task, children are asked to read aloud as many words and nonwords as possible from a list in 45 s. The total number of correctly read words is then noted. No children were excluded from the main experiment based on their TOWRE‐2 scores as is routinely done in studies with typical readers. This was because we wanted to include children with severe word reading difficulties and ensured that our experimental texts were appropriate for those with a reading age of 7 years. All standardised tests were also suitable for children with low reading ability. Children completed two versions (Form A and Form B) of the TOWRE‐2, one in Calibri typeface and one in Dyslexie typeface. The order in which these were completed was counterbalanced, as was the version‐typeface combination that each child saw. The TOWRE‐2 has good levels of reliability: Cronbach's *α* = .91 and .92 for words and nonwords respectively.

Children also completed the rapid letter naming subtest of the Comprehensive Test of Phonological Processing (CToPP; Wagner, Torgesen, Rashotte, & Pearson, 2013). In this task, children are asked to name aloud as quickly and as accurately as possible a series of letters in rows. Although we used only one of the two naming subtests, naming overall has good reliability: Cronbach's *α* = .92. Like the TOWRE‐2, we had two versions of the task, one in Calibri and one in Dyslexie typeface. In addition, the letters used in the task were manipulated. In one version, the regular condition, the same letters used in the original task were used (s, t, n, a, k, c). In the alternative version, the confusable condition, letters known to be commonly mixed up were deliberately chosen (b, d, p, q, o, e). This was done as the creators of Dyslexie typeface argue that the typeface is especially beneficial for these confusable letters which differ only in one or two elements. Children completed the task twice, once in one typeface and once in the other, and once the regular version and once the confusable version. Therefore, the design was not fully crossed but the order and combination of versions was fully counterbalanced.

The final standardised test administered was the British Picture Vocabulary Scale (BPVS‐II, Dunn, Dunn, Whetton, & Burley, 1997), a test of receptive vocabulary. In this test, children hear a word (spoken by the researcher) and see four pictures (line drawings). They are asked to point to the picture that best represents the word they have heard. The test comprises 144 items which are arranged within 12 blocks of increasing difficulty (12 items per block). Testing is discontinued when the child makes eight or more errors within a block. This test was administered once per child in line with manual instructions. Good reliability is reported in manual (median Cronbach's *α* = .93).

#### 2.2.1 Target words

The experimental texts were created in the following way. First 52 target words were chosen. Twenty‐six were classed as easy‐to‐read and 26 as hard‐to‐read. The difficulty manipulation was based on the order (known as *phases*) in which children are taught key letter sounds in the primary school curriculum in England (Primary National Literacy Strategy, 2007). These mostly related to the vowel sounds but consonants and consonant clusters were also considered. Words in the easy‐to‐read category contained phonemes that are taught in Phases 2 and 3 (out of six) and had the most common pronunciation for vowel phonemes (e.g., *beetle* and *snooze*). Hard‐to‐read words contained phonemes taught in Phase 5 (as well as those taught in Phases 2 and 3) and tended to have less‐common pronunciations of vowel phonemes (e.g., *eight* and *heart*) and included words on KS2 spelling lists which are considered “tricky” (e.g., *rhyming* and *queue*). In addition, 43 adults, mostly teachers, scored each word on a scale of 1–6 on how difficult they would be for children aged 8–12 years to read. Table 2 shows the mean scores which were significantly higher (i.e., harder) for the hard‐ than easy‐to‐read words, *t*(50) = 9.19, *p* < .001. Target words were matched on number of letters, number of phonemes, number of syllables, word frequency, age of acquisition, familiarity, and concreteness (see Table 2). It should be noted that word frequency was numerically higher in the hard‐to‐read condition but this was driven by one extreme score (great had a frequency of 820/million). Since it is known that once word frequencies rise above 50/million, there is little impact on word recognition times (Brysbaert, Mandera, & Keuleers, 2018), this was not of great concern.

**TABLE 2:** Characteristics of target words in the easy‐ and hard‐to read conditions

	Easy‐to‐read	Hard‐to‐read	*t*	*p*
Number of letters	5.8 (1.18)	6.0 (0.87)	.94	.35
Number of phonemes	4.6 (0.96)	4.4 (1.36)	.54	.56
Number of syllables	1.6 (0.57)	1.6 (0.64)	0	1
Frequency^a^	41.8 (73.5)	107.5 (192.9)	1.60	.12
AoA^a^	6.0 (2.16)	6.4 (1.94)	.81	.4
Familiarity^b^	529 (52.8)	550 (51.7)	1.16	.25
Concreteness^b^	479 (133)	473 (113)	.14	.89
Difficulty scoring (/6)^c^	2.2 (0.46)	3.6 (0.66)	9.19	<.001

*Note*: Columns show mean scores (standard deviations in parentheses), and *t*‐values and *p*‐values from a series of independent *t*‐tests comparing the two conditions.

^a^ Kuperman, Stadthagen‐Gonzalez, and Brysbaert (2012).

^b^ MRC psycholinguistic database, Coltheart (1981) (some values were missing).

^c^ Rating on a scale 1–6 on how difficult words were for children to spell.

#### 2.2.2 Experimental texts

Seven passages were then created in which target words could be embedded such that an easy‐to‐read word and its hard‐to‐read counterpart could be substituted within the same sentence. We did not want the target words to be obvious to the readers as we wanted them to read the passages as naturally as possible. Each passage contained 3–4 target words and the passages were all short, simple stories with short sentences and simple vocabulary and were appropriate for the reading level of the children in the study, as judged by readability measures of the texts (Table 3). All passages were followed by one simple comprehension question designed to maintain focus and check children were on task, rather than to assess comprehension. A full list of the experimental passages and the target words are provided in the Appendix.

**TABLE 3:** Mean readability measures for the experimental passages. Standard deviations in parentheses

Measure	Mean *(SD)*
Number of characters (without spaces)	487 *(28.6)*
Number of words	121 *(4.7)*
Number of sentences	9.7 *(2.20)*
Lexical density (ratio of content words to total number of words)	44.6 *(3.59)*
Number of characters per word	4.0 *(0.17)*
Number of syllables per word	1.4 *(0.06)*
Number of words per sentence	13.0 *(2.49)*
Gunning fog index (years of school needed to read text)	7.1 *(1.36)*
Flesch Kincaid grade level (grade level needed to read text)	5.4 *(0.91)*
Flesch Reading ease (/100)	79.3 *(4.48)*

### 2.3 Apparatus

Throughout passage reading, children's eye movements were recorded using Eyelink Portable Duo eye tracker (SR Research, Mississauga, Canada) as they read from a computer monitor at a viewing distance of approximately 50 cm. Eye movements were monitored at a rate of 1,000 Hz to produce a sequence of fixations with start and finish times. Although children read binocularly, only the movements of the right eye were monitored.

### 2.4 Procedure

All participants were seen individually at school, in a quiet room near to their classroom. First children completed both the word and nonword subtests of the TOWRE in either Calibri or Dyslexie typeface. This took approximately 3 min. Next, they completed the rapid naming letters subtest of the CToPP. This was done in Calibri or Dyslexie typeface and the letters were either those in the original test (regular) or those chosen to be high in overlapping features (confusable). This took less than 3 min.

Next participants did the main eye tracking experiment. For this, they were seated at the eye tracker, facing a computer monitor. There was a chin rest for comfort and to minimise head movements; however, they were not restrained in any way. After explaining the procedure to the children, they were calibrated. For this, they were asked to look at a series of nine fixation points that appeared on the computer monitor. For each trial they were asked to fixate a central fixation dot to ensure calibration remained accurate and then to fixate a small box at the top left of the screen. Upon fixating this box, the passage for the next trial was presented. This was to ensure that their initial fixation was at the start of the text. Children were told that they would read a series of short stories and that they should read normally (as they would a book). When they had finished reading the passage, they were asked to press either of two buttons on a handheld gamepad controller. If they did not do so, after 90 s, the display automatically terminated. They were also told that they would be asked a yes/no question after each story and that they answered this question by pressing one of two buttons on the gamepad controller (corresponding to yes and no). Paragraphs were presented in a pseudorandom order such that each child saw only one of the four versions of each stimulus set (Calibri or Dyslexie typeface, and easy‐to‐read or hard‐to‐read spellings). There was one practice passage (checking understanding of the instructions), followed by seven experimental passages. The eye tracking section lasted 10–15 min in total.

Next, the participants completed the BPVS. This took 10–12 min. They then repeated the TOWRE and the RAN, this time in the typeface, (and letter type for the RAN) that they had not already completed. Together these took about 6 min to complete. The child was then given a certificate and thanked for their time. The testing session took 40–50 min in total. While most children completed all tests in a single session, a small number of children came back to complete one or more tests on a subsequent day.

## 3 RESULTS

### 3.1 Offline tests

For the two offline tests, rapid naming and reading efficiency, data were analysed using SPSS 25 (IBM Corp, 2017).

#### 3.1.1 Rapid naming

Naming times for children who made more than five naming errors in total were excluded from the analysis. Table 4 shows the number of exclusions per condition (note each child completed two RAN tasks so 11 exclusions in total accounted for 7.7% of the data). It is clear from Table 4 that there were more exclusions in the confusable letters version of the task and that the dyslexia group were subjected to slightly more exclusions.

**TABLE 4:** Number of excluded data points in the RAN task per condition and group

	Control		Dyslexic	
	Dyslexie	Calibri	Dyslexie	Calibri
Regular	1	0	0	1
Confusable	2	2	3	2

**FIGURE 1:**
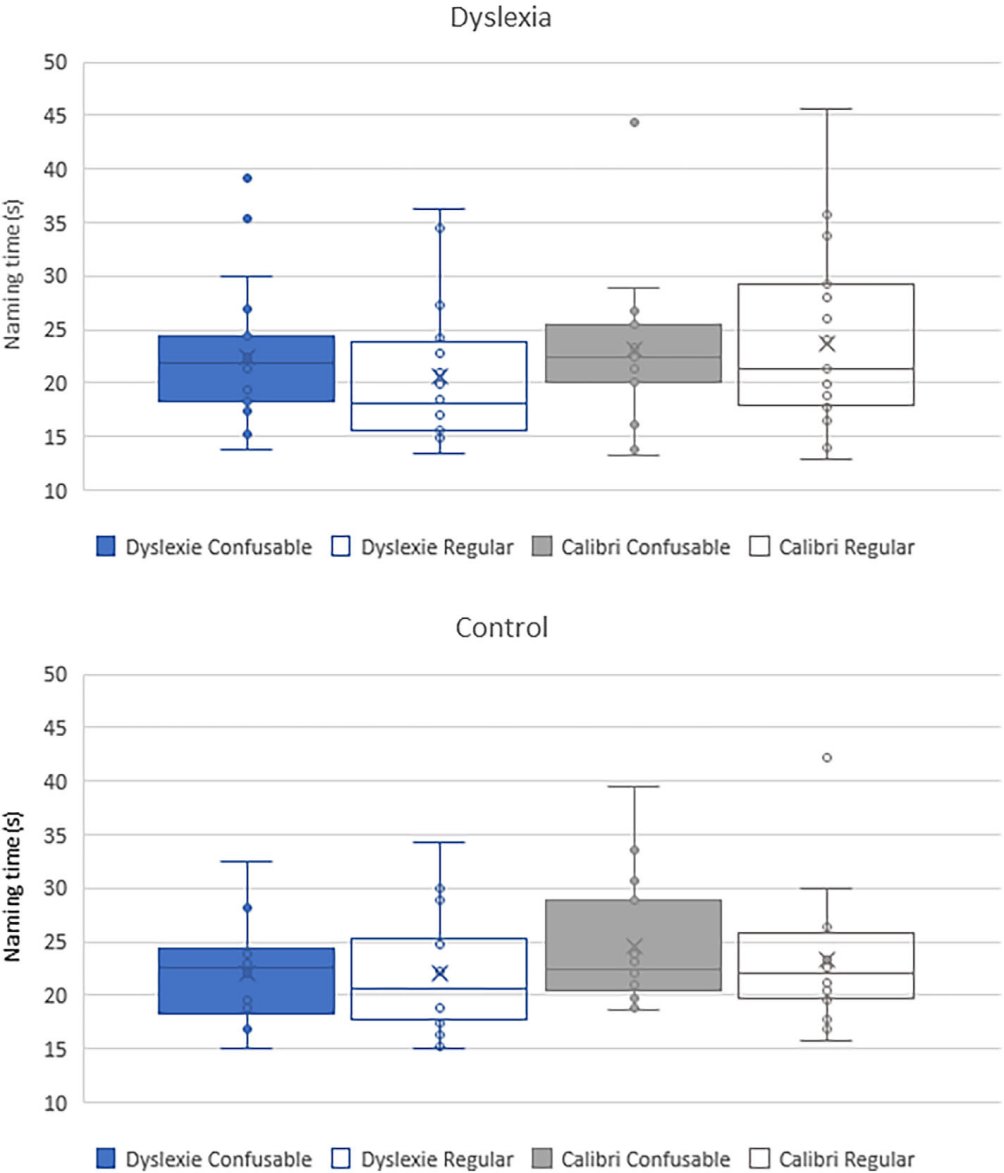
Mean naming times for children with (top panel) and without (bottom panel) dyslexia on letters in Dyslexie and Calibri types for regular and confusable letters

For the rapid naming task, we were interested in whether children were faster naming letters in Dyslexie than Calibri typeface and naming regular versus confusable letters, and whether this differed as a function of group (dyslexic versus control). Table 5 and Figure 1 shows mean naming times in both typefaces and both letter types for dyslexic and control groups.

**TABLE 5:** Mean naming times in seconds (*SD* in parentheses) for sets of regular and confusable letters in Dyslexie and Calibri typefaces for children with and without a diagnosis of dyslexia

		Control	Dyslexia
Regular	Dyslexie	21.82 *(5.43)*	20.54 *(6.47)*
	Calibri	24.22 *(5.42)*	23.47 *(7.13)*
Confusable	Dyslexie	22.02 *(4.60)*	23.16 *(6.30)*
	Calibri	26.23 *(6.17)*	23.73 *(8.23)*

Because we did not have a full Latin Square Design (participants did not complete all four versions of the RAN task), we first conducted independent t‐tests to examine whether there were naming time differences for confusable versus regular letters in the RAN letter naming task in both typefaces. Results showed no significant differences between confusable and regular letters in Calibri typeface, *t*(63) = .16, *p* = .88, *d* = 0.04, or in Dyslexie typeface, *t*(63) = .63, *p* = .53, *d* = 0.16. We then conducted a mixed ANOVA with group (dyslexic versus control) and typeface (Dyslexie versus Calibri) as factors which showed faster naming times in Dyslexie typeface, *F*(1, 59) = 5.22, *p* = .026, *ηp* ^2^ = 0.81, with a 2.2 second advantage for Dyslexie typeface. There was no effect of group, *F*(1, 59) = 0.0005, *p* = .98, *ηp* ^2^ = 0.000008, and no interaction between group and typeface, *F*(1, 59) = .552, *p* = .46, *ηp* ^2^ = 0.009.

#### 3.1.2 Reading efficiency

Table 6 shows mean raw scores for word and nonword reading efficiency in Calibri and Dyslexie typefaces for children with and without dyslexia. A mixed ANOVA revealed no effect of typeface, *F*(1, 68) = 2.07, *p* = .1, *ηp* ^2^ = 0.03, no effect of group, *F*(1, 68) = .162, *p* = .688, *ηp* ^2^ = 0.004, and no interaction, *F*(1, 68) = .249, *p* = .62, *ηp* ^2^ = 0.004, in word reading efficiency. A second ANOVA revealed the same pattern for nonword reading: no effect of typeface, *F*(1, 68) = .844, *p* = .32, *ηp* ^2^ = 0.012, no effect of group, *F*(1, 68) = 1.83, *p* = .18, *ηp* ^2^ = 0.0003, and no interaction, *F*(1, 68) = .018, *p* = .89, *ηp* ^2^ = 0.0003.

**TABLE 6:** Mean raw scores (Standard deviations in parentheses) for word and nonword reading efficiency in Dyslexie and Calibri typefaces for children with and without a diagnosis of dyslexia

		Control	Dyslexia
Words	Calibri	56.9 (13.2)	58.4 (14.1)
	Dyslexie	58.2 (12.7)	58.9 (12.9)
Nonwords	Calibri	30.6 (11.8)	26.8 (10.9)
	Dyslexie	31.4 (11.4)	27.3 (12.5)

### 3.2 Eye movement measures

Data were analysed in the R computing environment (R Core Team, 2022) using linear mixed‐effects models (Baayen, Davidson, & Bates, 2008; Jaeger, 2008). All models included random intercepts for participants and items and random by‐participant and by‐item slopes for all fixed effects (i.e., a full random slopes structure; see Barr, Levy, Scheepers, & Tily, 2013). All fixed effects were centred using contrast coding to reduce the effects of collinearity between the main effects and interactions and so that main effects were evaluated as the average effects over levels of the other predictors (i.e., the intercept of the model is the mean of all data points in the dataset). Regression coefficients, standard errors, and *t* values are reported. Following Vorstius et al. (2014), we used the two‐tailed criterion (*t* or *z* > 1.96 SE), corresponding to a 5% error criterion for significance for all tests. We adjusted for multiple comparisons in the target word analyses by dividing our alpha level by four (the number of dependent variables), while also accounting for a mean correlation between dependent variables of 6. This lowered our alpha level to .0125 (which corresponded to a *t* value of 2.57).

For all eye movement data, fixations shorter than 80 ms and longer than 1,200 ms were excluded, and trials that showed blinks or tracker loss on the target word were also deleted. As is common in eye movement studies, reading time data were not normally distributed and so were log transformed.

#### 3.2.1 Total passage reading time

First, we examined overall passage reading time (see Table 7). We calculated this simply as the total time spent reading each passage. Such a measure is in line with previous studies that have examined text reading in Dyslexie and other typefaces (e.g., Kuster et al., 2018). There was a main effect of group, *b = 1731, SE* = 795.80, *t* = 2.18: children with dyslexia read the passages more slowly overall. However, there was no effect of typeface (*b =* 36.49*, SE* = 792, *t* = 0.05) and no interaction between group and typeface (*b =* 1,184*, SE* = 1,121, *t* = 1.06).

**TABLE 7:** Mean passage reading times in milliseconds (*SD* in parentheses) in Dyslexie and Calibri typefaces for children with and without a diagnosis of dyslexia

	Control	Dyslexic
Calibri	13,090 (6479)	14,701 (6644)
Dyslexie	12,822 (7171)	15,821 (7523)

#### 3.2.2 Target word analyses

We report four measures: first fixation duration, single fixation duration, gaze duration and total reading time. First fixation duration is the duration of the first fixation on the target word. Single fixation duration is the duration of a fixation on the target word if only one fixation was made (if more than one fixation is made then no data are returned). Gaze duration is the sum of all fixations on the target word before the eyes move to another word (to the right or the left). Finally, total reading time is the sum of all fixations on a word including refixations (going back to re‐read). We expected to see early effects (i.e., during first pass reading of our target words) since any effects of typeface or difficulty should be seen in early word processing rather than in later measures that are thought to reflect higher level text integration processes (Clifton et al., Clifton Jr, Staub, & Rayner, 2007). Nevertheless, we analysed total reading times on the target words in order to see if any effects were long‐lasting.

Mean reading times for all eye movement measures in all conditions are shown in Table 8. Results of the models are shown in Table 9.

**TABLE 8:** Mean reading times on the target word in for easy‐to‐read and hard‐to‐read words in Dyslexie and Calibri typefaces for children with and without dyslexia

Group	Eye movement measure	Dyslexie typeface		Calibri typeface	
		Easy‐to‐read	Hard‐to‐read	Easy‐to‐read	Hard‐to‐read
Dyslexic	First fixation duration	307 *(139)*	313 *(167)*	323 *(188)*	320 *(188)*
	Single fixation duration	333 *(176)*	347 *(181)*	345 *(192)*	323 *(175)*
	Gaze duration	475 *(350)*	454 *(318)*	491 *(354)*	467 *(401)*
	Total reading time	672 *(552)*	687 *(540)*	743 *(591)*	755 *(662)*
Control	First fixation duration	323 *(184)*	344 *(190)*	362 *(192)*	326 *(192)*
	Single fixation duration	407 *(191)*	392 *(166)*	400 *(228)*	354 *(180)*
	Gaze duration	433 *(253)*	471 *(289)*	443 *(263)*	434 *(264)*
	Total reading time	670 *(423)*	617 *(400)*	616 *(407)*	627 *(445)*

*Notes*: Reading times are in milliseconds. *SD* are in parentheses.

**TABLE 9:** Results of models examining the effect of group, typeface, and difficulty on all eye movement measures

		Group (dyslexic vs. control)	Typeface (Dyslexie vs. Calibri)	Difficulty (easy‐to‐read vs. harder‐to‐read)	
Main effects	First fixation duration	*b* = .042, *SE* = .052, *t* = 0.83	*b* = .03, *SE* = .04, *t* = 0.92	*b* = .02, *SE* = .04, *t* = 0.33	
	Single fixation duration	*b* = .15, *SE* = .06, *t* = 2.68*	*b* = .04, *SE* = .05, *t* = 0.98	*b* = .02, *SE* = .04, *t* = 0.55	
	Gaze duration	*b* = .01, *SE* = .06, *t* = 0.16	*b* = .01, *SE* = .04, *t* = 0.20	*b* = .03, *SE* = .05, *t* = 0.54	
	Total reading time	*b* = .03, *SE* = .07, *t* = 0.36	*b* = .01, *SE* = .04, *t* = 0.20	*b* = .02, *SE* = .04, *t* = 0.46	

		Group * typeface	Group * difficulty	Typeface * difficulty	Group * typeface * difficulty
Interactions	First fixation duration	*b* = .04, *SE* = .06, *t* = 0.57	*b* = .01, *SE* = .06, *t* = 0.15	*b* = .10, *SE* = .07, *t* = 1.39	*b* = .17, *SE* = .12, *t* = 1.40
	Single fixation duration	*b* = .11, *SE* = .08, *t* = 1.32	*b* = .04, *SE* = .08, *t* = 0.48	*b* = .05, *SE* = .08, *t* = 0.64	*b* = .01, *SE* = .14, *t* = 0.09
	Gaze duration	*b* = .04, *SE* = .07, *t* = 0.60	*b* = .06, *SE* = .07, *t* = 0.92	*b* = .02 *SE* = .09, *t* = 0.29	*b* = .08, *SE* = .14, *t* = 0.56
	Total reading time	*b* = .15, *SE* = .07, *t* = 2.06	*b* = .05, *SE* = .07, *t* = 0.78	*b* = .01, *SE* = .07, *t* = 0.19	*b* = .08, *SE* = .13*, t* = 0.64

In first fixation durations, there were no effects of difficulty, typeface, or group. In single fixation duration, there was an effect of Group with longer fixations in the control than the dyslexic group, but no other effects. It was also noted that although single fixations were longer in the control group, they made fewer of them (*M* = 7.4) than those in the dyslexic group (*M* = 11.9). There were no effects in gaze durations or in total reading times.

## 4 DISCUSSION

The current study sought to add to the literature examining the possible benefit of using specialist typefaces with children with reading difficulties. Although evidence to date does not support the use of these, often commercially available, typefaces, they continue to be widely used by teachers, parents, and dyslexic readers in the belief that it will make reading more fluent and less arduous. Previous research has clearly shown that there is no advantage to specialist typeface designs once spacing has been controlled for (Marinus et al., 2016). However, it is possible that measuring overall reading time does not reveal subtle but potentially important differences in reading behaviour. It is also possible that the efficiency of letter identification is influenced by typeface while reading words and sentences is not. We therefore monitored children's eye movements as they read passages containing target words which were similar in length and frequency. In addition, we asked children to complete two standardised tests of rapid naming and word and nonword reading in both typefaces to examine whether either skill was affected by typeface design.

In general, our results do not provide evidence for a benefit for a specialist typeface, Dyslexie, as compared with a widely‐used typeface, Calibri in word or passage‐level reading. However, there were some effects that merit further investigation. We will discuss in detail below the interpretation of our results in relation to rapid naming, word and nonword reading, and eye movements during passage reading in turn. We will then turn to a discussion of the limitations and educational implications of this research and suggestions for future study.

### 4.1 Rapid naming

Performance on rapid naming (RAN) tasks has been shown across numerous studies to predict later reading in children with (and without) reading difficulties (Araújo, Reis, Petersson, & Faísca, 2014). Given the rationale behind many specialist typefaces, that the distinctiveness of the letters makes letter identification more efficient and less error‐prone, it is reasonable to predict that any advantage conferred by typeface design will be seen in this task. We administered two versions of the RAN task in two different typefaces to our participants: a version with the same letters as in the original version (s, t, n, a, k, c), and a version with letters that we judged to be confusable (d, p, o, b, q, e). Our results showed no difference between these two versions of the task. However, we did find a time benefit of 2.2 s for Dyslexie typeface. This did not differ across our two participant groups, suggesting that Dyslexie typeface helped all children to name the letters more efficiently.

This finding is in contrast with previous studies (Kuster et al., 2018; Marinus et al., 2016) showing no advantage for word or passage reading for Dyslexie versus Arial typeface. However, letter naming is of course quite different to reading words or sentences and it may be that any very subtle benefits of more distinctive letter shapes and design are observable only when the task requires a focus on the letters themselves. We know from previous research that readers do not fixate every letter, or even every word when reading connected text (Rayner, Chace, Slattery & Ashby, 2006) and so other factors such as word length and frequency as well as vocabulary knowledge and using previous context to predict upcoming words may overshadow any subtle effects of typeface design. It is also possible that the benefits of Dyslexie to letter identification were outweighed by competing top‐down influences at the word or even sentence level, given research showing evidence of such top‐down effects in developing readers with and without dyslexia (Coch et al., 2012; Grainger et al., 2003), as mentioned above. The process of learning to read is multifaceted and influenced by both cognitive and affective factors (Eklund, Torppa, Sulkunen, Niemi, & Ahonen, 2018; Guthrie, Wigfield, Metsala, & Cox, 1999). For a beginning reader, particularly one with phonological problems related to dyslexia, one might speculate that more distinctive letter forms provided by Dyslexie might provide a stronger anchor for developing grapheme‐phoneme correspondences and thus make the earliest stages less effortful and aversive than might otherwise be the case, with downstream benefits on reading which are not necessarily obvious in experimental research such as this. An intervention study with beginning readers might shed light on any potential developmental effects of such specialist typefaces.

### 4.2 Word and nonword reading

We observed no difference between typefaces for word and nonword reading efficiency and this was the case for both the control and the dyslexic group. Indeed, if anything there was a very small tendency towards faster reading (approximately one word faster) in Calibri typeface. This finding is in line with previous studies that looked at word reading (Kuster et al., 2018) and indicates that at the word level, typeface design does not affect reading efficiency in children with or without dyslexia and we now have evidence that this is the case with children as young as seven (Marinus et al., 2016) up to adolescence (current study).

### 4.3 Passage reading

Consistent with previous studies (Duranovic et al., 2018; Kuster et al., 2018; Marinus et al., 2016), we saw no advantage for Dyslexie typeface in terms of overall passage reading times, although the dyslexic group did read more slowly overall. There was a very small tendency for the dyslexic group to read faster when the typeface was Calibri and for the control group to read faster in Dyslexie but this was not significant and so cannot be taken as evidence of an educationally meaningful advantage. As in word reading, it may be that the complexity of the reading task that calls on a number of cognitive and linguistic skills and that small differences in letter shape cannot compete with these.

### 4.4 Eye movements during passage reading

Previous studies that have compared Dyslexie with a standard typeface in passage reading have examined overall passage reading time. In our study we wanted to examine passage reading at a more fine‐grained level. Each of our passages contained carefully selected target words which were matched for length, frequency, number of morphemes and syllables but manipulated for reading difficulty as related to stages of phonics teaching in UK schools. We found no effects of difficulty, and no main effects of typeface. We did find a main effect of group, such that the control group made longer single fixations than the dyslexic group on our target words. This was unexpected given that: (1) dyslexic readers tend to make longer fixations than typical readers (Ashby, Rayner, & Clifton, 2005); and (2) our two groups were matched on reading efficiency. Further examination of number of fixations revealed that the control group did not make longer fixations in general, just when they made a single fixation on the target word, and furthermore they made fewer of these types of fixations than their dyslexic peers. Again, this is somewhat surprising given that single fixations (hence fewer fixations overall) are usually associated with more skilled reading (e.g., Hyönä & Olson, 1995). It is the case that our dyslexic group were quite significantly older than our control group, due to being matched on word reading efficiency. Therefore, it was almost certainly the case that our dyslexic group had acquired more reading experience (due to more years of exposure but also perhaps due to increased exposure from reading interventions) and were perhaps more adept at targeting their fixations closer to the optimal viewing location (Aghababian & Nazir, 2000; McConkie, Kerr, Reddix, Zola, 1988) in a word thereby reducing the need for an additional fixation on the same word before moving on.

### 4.5 Limitations

While our findings raise some interesting possibilities regarding the role of specialist typefaces in letter identification, we recommend caution in interpreting our findings for three main reasons. First our two participant groups came from two distinct schools so they are likely to have differed in many ways other than their diagnostic status. Although our control group were broadly average in term of their language and literacy skills, because our dyslexic group were recruited from a specialist school, and had received a diagnosis, it is likely that they were from more advantaged socioeconomic backgrounds than average and from the control group (Macdonald & Deacon, 2019). They also received 30 min of one‐to‐one targeted literacy support a day, which a dyslexic child is unlikely to receive in a mainstream, non‐specialist setting.

Second, we should acknowledge that we did not have a fully crossed design for our RAN task, the only task in which we observed an effect. This reduced statistical power but more importantly meant that we were not able to carry out an omnibus ANOVA. Future studies should certainly address this limitation and our current results, though interesting, should be treated with caution.

Finally, the lack of effects of our difficulty manipulation in the passage reading task was unexpected and may suggest that other confounding factors were at play. Although we controlled our target words on a large number of lexical characteristics known to influence word processing, as the main manipulation was straightforward and a condition for observing any effect of typeface, it may be that we failed to consider other influences. It is possible that had our manipulation been more successful, a benefit of Dyslexie typeface may have been observed and it is worth taking this into account in future studies.

### 4.6 Practical implications and future research

One interesting question that emerges from the pattern of effects observed is whether novelty may play a role. No child reported having seen Dyslexie typeface prior to the experiment and so they were faced with the additional challenge of identifying letters whose exact form they had not yet encountered. While we know that skilled and less skilled readers are able to do this quite easily (Pelli, Burns, Farell, & Moore‐Page, 2006), it is also the case our perceptual systems can become “tuned” to a certain typeface over time (Sanocki, 1987). It may be then that the biggest potential benefit of a specialist typeface such as Dyslexie is in the very early stages of learning to read. We know that young children often confuse similar letters (Terepocki et al., 2002) and so distinctiveness at this stage when children have many new and overlapping visual symbols to commit to memory may reduce this phase of learning. It is unlikely that a specialist typeface will help with learning letter sound correspondences but if it is particularly the letters that are causing a child to struggle, then a specialist typeface may help. Future intervention research on the efficacy of using a specialist typeface as compared to those standardly used in nursery and pre‐school settings can reveal whether this could be one element of support put in place alongside a focus on phonological awareness and letter sound learning. This may be of particular benefit to children at risk of reading difficulties (Elbro, Borstrøm, & Petersen, 1998) due to a family history of dyslexia.

## 5 CONCLUSION

If unnoticed or unsupported, reading difficulties such as dyslexia can have a potentially devastating effect on a child's education and future life chances. It is therefore imperative that we continue to seek evidence‐based interventions to support all children to fulfil their potential. Easy to administer interventions such as using a specialist typeface are attractive as they offer the possibility of improved outcomes with relatively litte input, money, or effort. Although a number of studies, including this one, have shown that typeface design does not result in more fluent or accurate reading of words, sentences, or passages, the current data are consistent wi a possible advantage of typefaces with more distinctive letters such as Dyslexie for letter identification. While the current results should be treated with caution, they do suggest that an early years intervention in which children are taught letters and sounds in a specialist typeface is worthy of consideration.

## Data Availability

The data that support the findings of this study are available from the corresponding author upon reasonable request.

## APPENDIX A

Below are all experimental passages in full with target words in bold (these were not in bold in the experiment itself). Each passage has two versions which are presented separately. The comprehension question asked after each passage is also included.

**Passage 1.**

Version 1.

Billy and Paul were having lunch together on the last day of term. They were both from Spain and were good friends, maybe because they spoke the same **language** and they both loved football. They were picking their food at the counter when they saw a dish they did not recognise. Billy said he thought the food was **common** although he'd never seen it before. They decided to be brave and try it. They both asked for a helping of it and were surprised by the **weight** when they were served. Just as they were about to tuck in, they were told that there was a **leak** in the kitchen. They left the new dish untouched and ran in to see it.

Version 2.

Billy and Paul were having lunch together on the last day of term. They were both from Spain and were good friends, maybe because they liked the same **sandwich** and they both loved football. They were picking their food at the counter when they saw a dish they did not recognise. Billy said he thought the food was **foreign** although he'd never seen it before. They decided to be brave and try it. They both asked for a helping of it and were surprised by the **plate** when they were served. Just as they were about to tuck in, they were told that there was a **steak** in the kitchen. They left the new dish untouched and ran in to see it.

Question.

Did Billy and Paul try the new dish?

**Passage 2.**

Version 1.

As the children came out of the forest, they could see a beautiful **rainbow** in front of them. Emily thought she could see a **giraffe** in the distance although she wasn't sure. They needed to hurry home. As they walked further, they saw a **beetle** whizz past them at top speed. “Wow, that was fast!” exclaimed Suzie. At last they came to a building which looked like a farmhouse. There were lots of animals and a table set for breakfast outside. “Oh what a lovely **pretty** cat!” cried Emily as she stroked its head. It purred happily. The **bunny** on the table looked lovely and Suzie wanted to bring it with them. But they had to continue on their way or they would not be home before dark.

Version 2.

As the children came out of the forest, they could see a beautiful **meadow** in front of them. Emily thought she could see an **aircraft** in the distance although she wasn't sure. They needed to hurry home. As they walked further, they saw a **vehicle** whizz past them at top speed. “Wow, that was fast!” exclaimed Suzie. At last they came to a building which looked like a farmhouse. There were lots of animals and a table set for breakfast outside. “Oh what a lovely **kitty** cat!” cried Emily as she stroked its head. It purred happily. The **honey** on the table looked lovely and Suzie wanted to bring it with them. But they had to continue on their way or they would not be home before dark.

Question.

Did Suzie take anything with her on the way home?

**Passage 3.**

Version 1.

It was Billy and Amanda's first day at secondary school and he was showing off his new **friend** in the school hall. He wanted the other children to look and stare. Billy asked Amanda about her hobbies. Billy really loved **flying** and hoped that Amanda did too. But Amanda said she preferred reading books and dancing. As they waited for the bell to ring they looked up at the sky and saw an army helicopter. What a **height** it was: they could hardly believe it. As it flew out of sight, Billy and Amanda looked at each other. It was time to join the **crew** at the classroom door: lessons were about to start.

Version 2.

It was Billy and Amanda's first day at secondary school and he was showing off his new **trend** in the school hall. He wanted the other children to look and stare. Billy asked Amanda about her hobbies. Billy really loved **science** and hoped that Amanda did too. But Amanda said she preferred reading books and dancing. As they waited for the bell to ring they looked up at the sky and saw an army helicopter. What a **surprise** it was: they could hardly believe it. As it flew out of sight, Billy and Amanda looked at each other. It was time to join the **queue** at the classroom door: lessons were about to start.

Question.

Did Amanda like reading books and dancing?

**Passage 4.**

Version 1.

Mr Robson came out of the **theatre** at half past five and looked at his watch. He was a businessman and had some important decisions to make. He did not want to sell his companies but he had to do something. He knew his **freedom** was the most important thing. He did not want to owe other people money all his life just for the sake of keeping his job. There was a **danger** that he had to consider as well. Many people's jobs depended on the decisions he made today. He decided to go home, talk to his wife, and think about it some more. Some decisions are too important to rush.

Version 2.

Mr Robson came out of the **metro** at half past five and looked at his watch. He was a businessman and had some important decisions to make. He did not want to sell his companies but he had to do something. He knew his **museum** was the most important thing. He did not want to owe other people money all his life just for the sake of keeping his job. There was a **painter** that he had to consider as well. Many people's jobs depended on the decisions he made today. He decided to go home, talk to his wife, and think about it some more. Some decisions are too important to rush.

Question.

Did Mr Robson make his decision straight away?

**Passage 5.**

Today was the day Emily and Suzie had been waiting for: the cross country running race. They had decided to meet at the **skate** park in the centre of town so that they would have plenty of time to get there. Just before the race, Emily worried about her **heart** as she was so nervous. But she told herself to be calm and that she could do it, and that seemed to help. Suzie had hurt her **bottom** when she had fallen down the week before but she felt OK now. As the bell rang for the race to start, the girls began to run. “It would be so **great** if we won this race.” said Emily to Suzie as they ran through the park.

Version 2.

Today was the day Emily and Suzie had been waiting for: the cross country running race. They had decided to meet at **eight** o'clock in the centre of town so that they would have plenty of time to get there. Just before the race, Emily worried about her **start** as she was so nervous. But she told herself to be calm and that she could do it, and that seemed to help. Suzie had hurt her **muscle** when she had fallen down the week before but she felt OK now. As the bell rang for the race to start, the girls began to run. “It would be so **cool** if we won this race.” said Emily to Suzie as they ran through the park.

Question.

Was Emily nervous before the race?

**Passage 6.**

Version 1.

Chloe and Sam were mad about tennis. They loved playing the **sport** and played almost every weekend. Sometimes they would sing their special counting song which helped them to keep hitting the ball back to each other. They were not too sure about the **rhyming** but did the best they could. One day they had been playing for about an hour when Sam let out a cry of pain. “What's wrong?” asked Chloe. “I'm not sure,” replied Sam, “But I think I'm so tired that I hit myself with my racket!”. “Wow, you must be really tired,” replied Chloe. “Yes, I think I'll have a **snooze** later,” said Sam. “You can have too much of a good thing!”

Version 2.

Chloe and Sam were mad about tennis. They loved playing on the court and played almost every weekend. Sometimes they would sing their special counting song which helped them to keep hitting the ball back to each other. They were not too sure about the **timing** but did the best they could. One day they had been playing for about an hour when Sam let out a cry of pain. “What's wrong?” asked Chloe. “I'm not sure,” replied Sam, “But I think I'm so tired that I hit myself with my racket!”. “Wow, you must be really tired,” replied Chloe. “Yes, I think I'll have a **bruise** later,” said Sam. “You can have too much of a good thing!”

Question.

Was football Chloe and Sam's favourite sport?

**Passage 7.**

Version 1.

Sam and Chloe were twins and always sat next to each other in class. During spelling tests, they sometimes copied each other if the teacher wasn't looking. On Friday, they had a test and Chloe did not know the first spelling. Sam wrote the word **century** in his book and then lifted the page slightly so Chloe could see. Sam was good at spelling and Chloe was good at Maths so it worked very well. In Maths, sometimes Chloe distracted him with nice pictures and sometimes she tried to help him learn. On Monday she drew a big **scarf** and a tiny **circle** in her exercise book. Sam liked both types of pictures and it made him look forward to Maths lessons.

Version 2.

Sam and Chloe were twins and always sat next to each other in class. During spelling tests, they sometimes copied each other if the teacher wasn't looking. On Friday, they had a test and Chloe did not know the first spelling. Sam wrote the word **celery** in his book and then lifted the page slightly so Chloe could see. Sam was good at spelling and Chloe was good at Maths so it worked very well. In Maths, sometimes Chloe distracted him with nice pictures and sometimes she tried to help him learn. On Monday she drew a big **graph** and a tiny **turtle** in her exercise book. Sam liked both types of pictures and it made him look forward to Maths lessons.

Question.

Did Sam and Chloe help each other in lessons?
